# Correction to: Informed consent and informed intervention: SARS‑CoV‑2 vaccinations not just call for disclosure of newly emerging safety data but also for hypothesis generation and testing

**DOI:** 10.1186/s40001-021-00574-y

**Published:** 2021-09-13

**Authors:** Johannes C. Fischer, Albrecht G. Schmidt, Edwin Bölke, Verena Keitel, Torsten Feldt, Björn Jensen, Noemi F. Freise, Dieter Häussinger, E. Marion Schneider, Derik Hermsen, Detlef Kindgen-Milles, Wolfram Trudo Knoefel, Jan Haussmann, Balint Tamaskovics, Christian Plettenberg, Kathrin Scheckenbach, Stefanie Corradini, Jutta Rox, Vera Balz, Kitti Maas, Livia Schmidt, Olaf Grebe, Anja Ehrhardt, Peter Arne Gerber, Matthias Peiper, Bettina Alexandra Buhren, Artur Lichtenberg, Amir Rezazadeh, Wilfried Budach, Christiane Matuschek

**Affiliations:** 1 Institute for Transplantation Diagnostics and Cell Therapeutics, Medical Faculty, University Hospital Dusseldorf, Heinrich-Heine-University, 40225 Düsseldorf, Germany; 2 Department of Radiation Oncology, Medical Faculty, University Hospital Dusseldorf, Heinrich-Heine-University Dusseldorf, Moorenstr. 5, 40225 Düsseldorf, Germany; 3 Department of Gastroenterology, Hepatology and Infectious Diseases, Medical Faculty, University Hospital Dusseldorf, Heinrich-Heine-University Dusseldorf, Moorenstr. 5, 40225 Düsseldorf, Germany; 4 Division of Experimental Anesthesiology, University Hospital Ulm, Ulm, Germany; 5 Central Institute for Laboratory Diagnostics and Clinical Chemistry, Medical Faculty, Heinrich-Heine University, Düsseldorf, Germany; 6 Department of Anesthesiology, Medical Faculty, Heinrich Heine University, Dusseldorf, Germany; 7 Department of Surgery and Interdisciplinary Surgical Intensive Care Unit, Medical Faculty, Heinrich Heine University, Düsseldorf, Germany; 8 Medicine, Department cBITE, Maastricht, The Netherlands; 9 Department of Otolaryngology–Head and Neck Surgery, Medical Faculty, Heinrich Heine University, Düsseldorf, Germany; 10 Department of Radiation Oncology, University Hospital, LMU Munich, Munich, Germany; 11 Department of Cardiology and Rhythmology, Petrus Hospital, Wuppertal, Germany; 12 Institute of Virology, University of Witten/Herdecke, Witten, Germany; 13 Medical Faculty, University of Dusseldorf, Düsseldorf, Germany; 14 Department of Cardiac Surgery, Medical Faculty, University of Dusseldorf, Düsseldorf, Germany

## Correction to: Eur J Med Res (2021) 26:87 10.1186/s40001-021-00558-y

In the original publication of the article [1], the author name Anja Ehrhardt was incorrectly written as Anja Erhardt.

The original article has been corrected.
